# Effects of diabetes mellitus and systemic arterial hypertension on elderly patients’ hearing^☆^^☆☆^

**DOI:** 10.1016/j.bjorl.2017.08.014

**Published:** 2017-09-21

**Authors:** Laurie Penha Rolim, Alessandra Giannella Samelli, Renata Rodrigues Moreira, Carla Gentile Matas, Itamar de Souza Santos, Isabela Martins Bensenor, Paulo Andrade Lotufo

**Affiliations:** a Universidade de São Paulo (USP), Faculdade de Medicina (FM), Departamento de Fisioterapia, Fonoaudiologia e Terapia Ocupacional, São Paulo, SP, Brazil; b Universidade de São Paulo (USP), Hospital Universitário (HU), São Paulo, SP, Brazil

**Keywords:** Hearing, Diabetes mellitus, Systemic arterial hypertension, Hearing loss, Elderly, Audição, Diabetes *mellitus*, Hipertensão arterial sistêmica, Perda auditiva, Idoso

## Abstract

**Introduction:**

Chronic diseases can act as an accelerating factor in the auditory system degeneration. Studies on the association between presbycusis and diabetes mellitus and systemic arterial hypertension have shown controversial conclusions.

**Objective:**

To compare the initial audiometry (A1) with a subsequent audiometry (A2) performed after a 3 to 4-year interval in a population of elderly patients with diabetes mellitus and/or systemic arterial hypertension, to verify whether hearing loss in these groups is more accelerated when compared to controls without these clinical conditions.

**Methods:**

100 elderly individuals participated in this study. For the auditory threshold assessment, a previous complete audiological evaluation (A1) and a new audiological evaluation (A2) performed 3–4 years after the first one was utilized. The participants were divided into four groups: 20 individuals in the diabetes mellitus group, 20 individuals in the systemic arterial hypertension group, 20 individuals in the diabetes mellitus/systemic arterial hypertension group and 40 individuals in the control group, matching them with each study group, according to age and gender. ANOVA and Kruskal–Wallis statistical tests were used, with a significance level set at 0.05.

**Results:**

When comparing the mean annual increase in the auditory thresholds of the A1 with the A2 assessment, considering each study group and its respective control, it can be observed that there was no statistically significant difference for any of the frequencies for the diabetes mellitus group; for the systemic arterial hypertension group, significant differences were observed after 4 kHz. For the diabetes mellitus and systemic arterial hypertension group, significant differences were observed at the frequencies of 500, 2 kHz, 3 kHz and 8 kHz.

**Conclusion:**

It was observed that the systemic arterial hypertension group showed the greatest decrease in auditory thresholds in the studied segment when compared to the other groups, suggesting that among the three studied conditions, hypertension seems to have the greatest influence on hearing.

## Resumo

**Introdução:**

Doenças crônicas podem atuar como fator de aceleração na degeneração do sistema auditivo. Os estudos sobre a associação da presbiacusia com o diabetes *mellitus* e com a hipertensão arterial sistêmica mostraram conclusões controversas.

**Objetivo:**

Comparar a audiometria inicial (A1) com uma audiometria sequencial (A2) feita com um intervalo de três a quatro anos em uma população de idosos portadores de diabetes mellitus e/ ou hipertensão arterial sistêmica, a fim de saber se a perda de acuidade auditiva nesses grupos é mais acelerada comparada com controles sem essas condições clínicas.

**Método:**

Participaram deste estudo 100 idosos. Para a análise dos limiares auditivos, foram usadas: uma avaliação audiológica completa feita anteriormente (A1) e uma nova avaliação audiológica (A2) feita após três a quatro anos da primeira. Os participantes foram distribuídos em quatro grupos: 20 indivíduos no grupo com diabetes mellitus, 20 no grupo hipertensão arterial sistêmica, 20 no grupo diabetes mellitus/hipertensão arterial sistêmica e 40 indivíduos no grupo controle, foram pareados com cada grupo de estudo, de acordo com as características referentes a idade e sexo. Foram usados os testes estatísticos Anova e Kruskal-Wallis, com nível de significância de 0,05.

**Resultados:**

Na comparação da média de aumento anual dos limiares auditivos da avaliação A1 com a avaliação A2, considerando cada grupo estudo e seu respectivo controle, pode-se observar que para o grupo diabetes mellitus não houve diferença estatisticamente significante para qualquer das frequências; para o grupo hipertensão arterial sistêmica foram observadas diferenças significantes a partir de 4 kHz. Já para o grupo diabetes mellitus/hipertensão arterial sistêmica foram observadas diferenças significantes nas frequências de 500, 2k, 3k e 8 kHz.

**Conclusão:**

Verificou-se que o grupo hipertensão arterial sistêmica foi o que apresentou maior queda dos limiares auditivos no segmento estudado, quando comparado com os outros grupos, sugeriu que entre as três condições estudadas a hipertensão parece ser a que teve maior influência sobre a audição.

## Introduction

The aging of the world's population is currently a worldwide phenomenon. Aging is related to the process of progressive degeneration and cell death, which leads to a decrease in the body's functional capacity.^1^, ^2^

The hearing loss resulting from the degenerative aging processes is known as presbycusis.^3^ It is currently the most frequent sensory impairment observed in the elderly, with a prevalence ranging from 25% in the 70–74 age group, 50% in the elderly aged up to 85 years, and greater than 80% in people over 85 years of age.^4^, ^5^

Presbycusis can cause a reduction in speech perception, psychological changes (such as depression), social isolation, problems related to alertness and defense (ability to hear automotive horns, telephone rings, alarms, etc.), as well as cognitive functions.^5^, ^6^ All these factors have a negative reflect on the elderly's quality of life.^5^, ^6^, ^7^

Despite the high prevalence of presbycusis, some authors have shown that the chronic diseases that most frequently affect the elderly, such as systemic arterial hypertension (SAH)^8^, ^9^, ^10^, ^11^, ^12^ and diabetes mellitus (DM)^13^, ^14^, ^15^, ^16^, ^17^, ^18^, ^19^ may be related with hearing impairment.

DM is a metabolic disease that causes vascular complications and neurological impairment. The number of adults with diabetes worldwide increased from 108 million in 1980 to 422 million in 2014.^20^ ELSA-Brazil, a cohort study of 15,105 civil employees aged 35–74 years, found a prevalence of 19.7% of diabetes.^21^ Hearing loss in individuals with DM may be related to diabetic angiopathy, which interferes with the supply of nutrients and oxygen to the cochlea, leading to cell and tissue death.^13^, ^22^, ^23^, ^24^ In addition to cochlear alterations, it is believed that DM can cause secondary degeneration of eighth cranial nerve fibers, resulting in neural hearing loss.^13^, ^23^, ^25^

Systemic arterial hypertension (SAH) is a multifactorial condition characterized by the presence of elevated blood pressure, associated with metabolic and hormonal changes and trophic phenomena (cardiac and vascular hypertrophy).^26^ According to a World Health Organization study carried out in 2013, the prevalence of SAH in adults older than 25 years of age is around 35% in the Americas.^27^ The aforementioned ELSA-Brazil study, carried out in 2015, identified a prevalence of 35.8% among the 15,103 assessed individuals.^28^ The impairment of oxygen and nutrient transport to the cells due to decreased capillary blood flow are among the pathogenic mechanisms of SAH that may be involved in hearing loss.^29^, ^30^ High blood pressure in the vascular system may result in bleeding in the inner ear, which may lead to permanent hearing loss.^31^ SAH can also cause hearing loss due to ionic modifications in cell potentials.^9^

Therefore, despite several studies on this topic concerning both DM and hypertension, there is still no consensus in the literature about the association between these alterations and hearing loss, especially in elderly individuals. The hypothesis of this study is that elderly hypertensive and/or diabetic patients show a more pronounced progressive hearing loss (during a 3-to-4-year follow-up) compared to individuals without these clinical conditions.

## Methods

The project was approved by the Ethics Committee of the institution under number 458.284.

### Sample

The ELSA-Brazil (Longitudinal Adult Health Study) was a multicenter cohort study of 15,000 employees from six public institutions of higher education and research in the Northeast, South and Southeast regions of Brazil. The research aimed to investigate the incidence and risk factors for chronic diseases, specifically cardiovascular diseases and diabetes. All active and retired staff and teachers aged 35–74 were eligible for the study.^32^, ^33^

**Figure 1:**
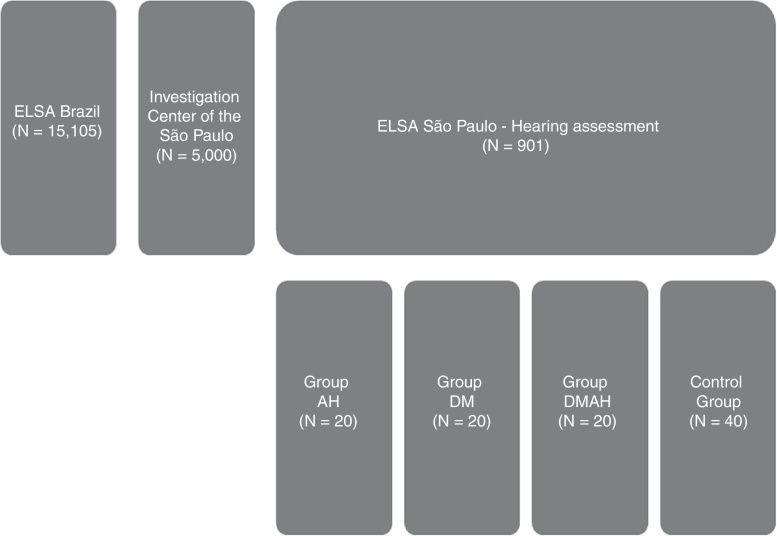
Flowchart of the selection steps. ELSA, Longitudinal Adult Health Study (*Estudo Longitudinal de Saúde do Adulto*); AH, arterial hypertension; DM, diabetes mellitus; DMAH, diabetes mellitus and arterial hypertension. ^a^Among the 40 individuals without SAH or DM, for each pairing (with the AH group or with the DM group or the DMAH group), 20 “healthy” participants were chosen, as described in the Data Analysis item.

This is an excerpt from the longitudinal hearing follow-up study of the ELSA-Brazil participants^34^ (Fig. 1). One hundred individuals participated in this study. For the auditory threshold assessments, a previous complete audiological evaluation (A1) and a new audiological evaluation (A2) performed after 3–4 years after the first one were utilized. The complete audiological evaluations consisted of: anamnesis, meatoscopy, immittance measurements, pure tone audiometry and speech audiometry.

The study inclusion criteria comprised the following: being 60 years of age or older; absence of external acoustic meatus (EAC) obstruction in both ears; absence of alterations in the middle ear demonstrated by immittance measurements; no history of occupational exposure to noise; having a complete initial audiological evaluation (A1), having diabetes mellitus for inclusion in the DM group; having systemic arterial hypertension for inclusion in the AH group; and DM associated with SAH for inclusion in the DM + AH group.

For inclusion in each of the groups, we relied on previous medical examinations performed in the same hospital. Diabetes was defined as use of medication to treat diabetes; fasting glycemia ≥ 126 mg/dL, glycated hemoglobin (HbA1c) ≥ 6.5% and/or 2-hour oral glucose tolerance test ≥ 200 mg/dL. Hypertension was defined as use of medication to treat hypertension; systolic blood pressure ≥ 140 mmHg or diastolic blood pressure ≥ 90 mmHg.

Thus, according to these criteria, the participants comprised four groups: 20 individuals with DM (DM group), 20 individuals with SAH (AH group), 20 individuals with DM and SAH (DMAH group) and 40 individuals with no DM or SAH (control group – CG).

### Materials and procedures

After the signing of the free and informed consent form, the following A2 audiological assessment procedures were performed: audiological anamnesis, meatoscopy, tympanometry with ipsilateral acoustic reflex assessment (the latter was performed only to verify the presence of middle ear alteration) and tonal audiometry at the frequencies of 250–8000 Hz and, if necessary, at the frequencies of 500–4000 Hz (when air conduction hearing threshold were worse than 20 dBHL). It is noteworthy that the A2 audiological assessment was performed 3–4 years after the A1 audiological assessment, following the wave periodicity of the ELSA-Brazil study.^32^, ^33^

### Data analysis

To study the influence of diabetes, hypertension or diabetes associated with hypertension in the evolution of auditory thresholds during the follow-up (3–4 years), participants with these clinical conditions were paired in a 1:1 ratio with participants of the same gender and age, in which these clinical conditions were absent. At each pairing, 20 “healthy” participants were selected from among the 40 subjects without SAH or DM, previously described as “control group”, using the Match function of the statistical software R Matching package. It was established that gender pairing should be exact for each participant, and that the age pairing would be chosen by the best approximation possible for the group. As a result, we obtained a perfect pairing for the distribution by gender and quite similar means for age in each of the groups compared to their control (maximum difference −0.25 years), thus allowing an adequate pairing.

To compare the auditory thresholds, since no statistically significant differences were observed between the ears for any of the studied groups, these were grouped. To compare the auditory thresholds of the first A1 assessment with the second A2 assessment between the study groups and their respective control, we considered the mean increase in auditory thresholds per year (considering the 3 or 4-year interval, depending on the date of reassessment of each individual).

In the statistical analysis, in addition to the descriptive measures, we used ANOVA and Kruskal–Wallis statistical tests, with a significance level of 0.05.

## Results

Table 1 shows the mean age of participants in each group. It can be observed that there was no statistically significant difference. The difference in mean age for each group and their respective control was very low (0.25 years in the worst case). The groups were also precisely paired for gender.

**Table 1:** Descriptive statistics and age comparison (in years) between the groups.

Groups	*n*	Gender	Mean age (SD)	*p*-Value
DM	20	12 F/8 M	64.05 (6.08)	1.00
CGDM	20	12 F/8 M	64.15 (5.89)	
AH	20	7 F/13 M	65.02 (4.33)	0.88
CGAH	20	7 F/13 M	65 (4.21)	
DMAH	20	11 F/9 M	64.25 (6.35)	0.88
CGDMAH	20	11 F/9 M	64.5 (6.37)	

F, female; M, male; SD, standard deviation; DM, diabetes mellitus; AH, arterial hypertension; CG, control group.

Regarding the age at diagnosis of hypertension and DM, in the DM group, the mean age was 61.75 years. In the AH group, the mean age was 52.65 years and in the DMAH group, the mean age at DM diagnosis was 58 years and for the SAH, it was 53.15 years. Regarding the time of the pathology diagnosis, in the DM group the mean number of years was 6.1. In the AH group, the mean was 16.6 years and in the DMAH group, the mean time of diagnosis for DM was 10.05 years and for SAH, 14.09 years.

**Figure 2:**
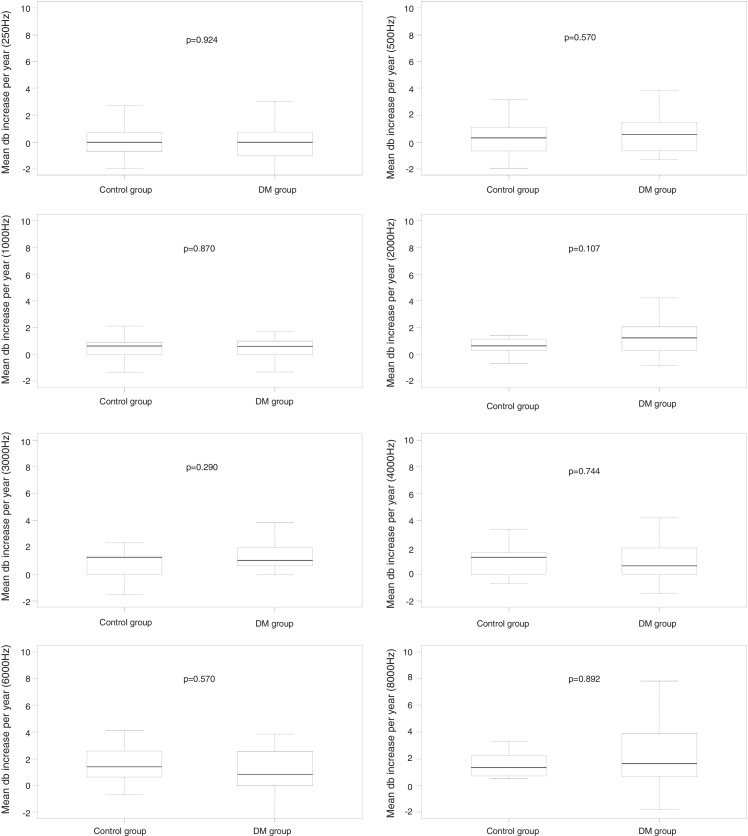
Comparison of the mean annual increase in auditory thresholds (in dBHL) at frequencies from 250 Hz to 8000 Hz between A1 and A2 assessments for the DM group and its respective control.

**Figure 3:**
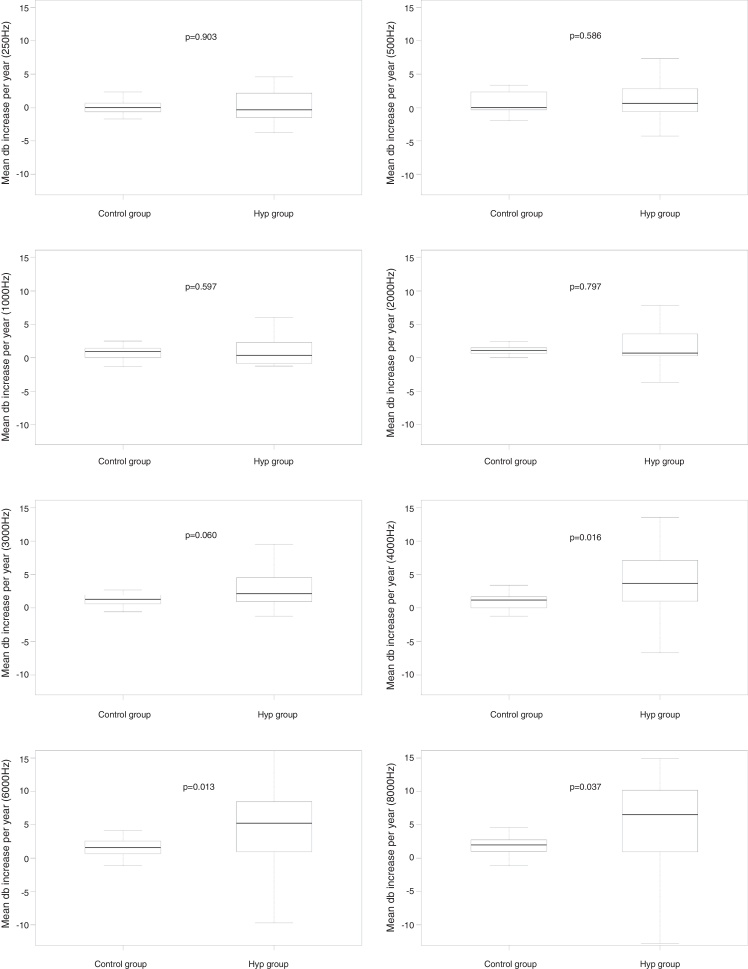
Comparison of the mean annual increase in auditory thresholds (in dBHL) at frequencies from 250 Hz to 8000 Hz between A1 and A2 assessments, for the AH group and its respective control.

**Figure 4:**
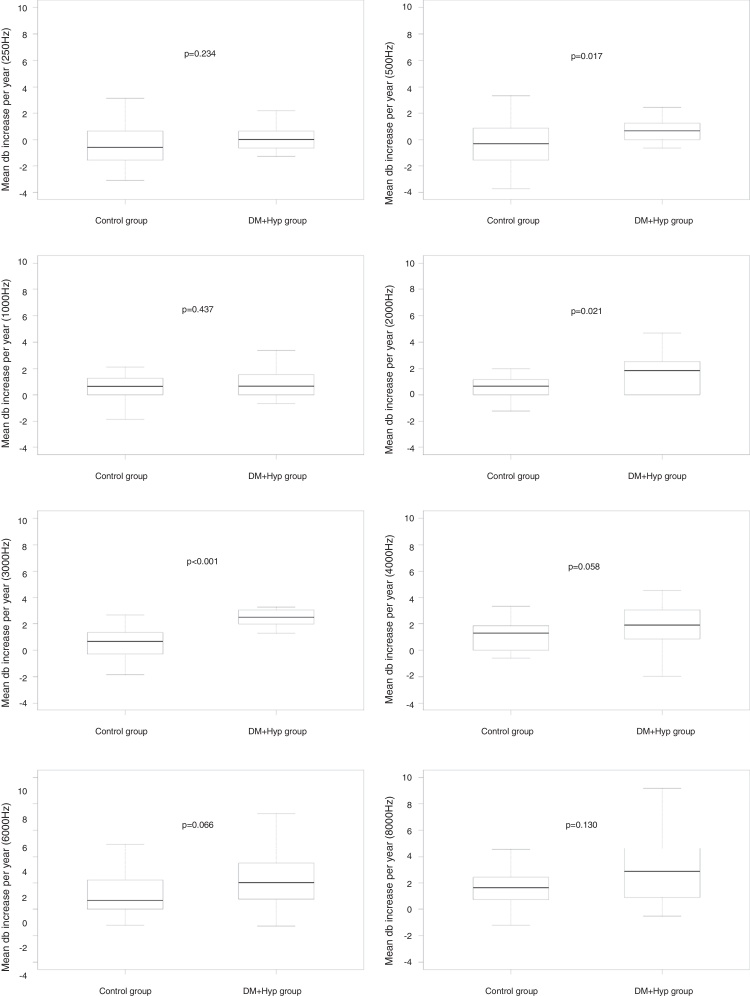
Comparison of the mean annual increase in auditory thresholds (in dBHL) at the frequencies from 250 Hz to 8000 Hz between the A1 and A2 assessments, for the DMAH group and its respective control.

When comparing the mean auditory thresholds at the first A1 assessment with the second A2 assessment between the groups, considering the mean increase in auditory thresholds per year, it can be observed that there was no statistically significant difference at any frequency for the DM group compared to its control group (Fig. 2); for the AH group, significant differences were observed at 4 kHz (*p* = 0.016); 6 kHz (*p* = 0.013), and 8 kHz (*p* = 0.037) compared to its CG, as well as a non-significant difference at 3 kHz (*p* = 0.060) (Fig. 3); for the DMHA group, significant differences were observed at the frequencies of 500 Hz (*p* = 0.017), 2 kHz (*p* = 0.021) and 3 kHz (*p* < 0.001) between the study group and its control, as well as non-significant differences at 4 kHz (*p* = 0.058) and 6 kHz (*p* = 0.066) (Fig. 4).

## Discussion

The study of conditions that may potentially influence the evolution of auditory function is important, since the earlier such changes are detected, the greater the chances of beneficial hearing rehabilitation. Thus, the aim of the present study was to compare the initial audiometry (A1) with a subsequent audiometry (A2) performed within a 3 to 4-year interval in a population of elderly individuals with DM and/or SAH, to assess whether the loss of auditory acuity in these groups is more accelerated compared to controls without these clinical conditions. Participants were paired for age and gender to minimize the effect of these variables on this association. Our findings indicated that the AH group showed the highest decrease in thresholds in the 3 to 4-year follow-up, followed by the DMAH group when compared to their control groups. The DM group did not show any significant differences in relation to its control group.

Some previous studies on the subject,^11^, ^16^ similar to the present study, sought to minimize the influence of the variables age and gender on the results of the audiological assessment, since it is known that these variables (mainly age) can affect auditory thresholds.^35^ A study carried out in Iran evaluated the hearing of 50 diabetic patients and 50 healthy subjects, paired by gender and age, and reported that hearing loss was more pronounced in the diabetes group, and speech discrimination was better in normal individuals. Esparza et al.^11^ compared the hearing of hypertensive and non-hypertensive individuals paired by gender and age, with ages ranging from 30 to 62 years, and subdivided the subjects into two groups, those with and without SAH. The authors observed greater cochlear dysfunctions in individuals with SAH and suggested it might be related to vascular disease from SAH.

When we compared the auditory thresholds from the first A1 assessment to those of the second A2 assessment between the study groups and their respective CGs, there was no statistically significant difference in the mean auditory threshold increase per year for any evaluated frequency in the DM group compared to its respective CG paired for gender and age.

Several authors have found a positive association between the presence of diabetes and auditory impairment,^13^, ^16^, ^36^ while others have not verified this association.^37^, ^38^ Some authors have suggested that this controversy may be related to the presence of many confounding variables, as well as the complexity of the auditory system.^34^, ^39^, ^40^

We observed that some studies that investigated the effect of diabetes on hearing did not exclude some of the confounding factors, such as: gender,^40^, ^41^, ^42^ age,^41^ and presence of arterial hypertension,^35^, ^39^ which may have influenced the observed results. Samelli et al.^34^ assessed hearing in 191 diabetic and 710 non-diabetic individuals, adults and elderly from ELSA-Brazil study (São Paulo); the authors did not find any statistically significant differences between the auditory thresholds of the two groups, after adjusting for age, gender and presence of hypertension, suggesting that these factors should be considered in studies of this kind. It is noteworthy that this study assessed cross-sectional data, while the present study used longitudinal data.

Regarding the AH group, considering the mean increase in auditory thresholds per year, significant differences were observed after 4 kHz, when this group was compared to its CG, as well as a trend to a statistically significant difference at 3 kHz. Therefore, we observed that the AH group had significantly worse auditory thresholds compared to non-hypertensive individuals paired by gender and age.

These findings agree with previous studies, which also verified more impaired auditory thresholds in individuals with SAH when compared to controls without this disease^8^, ^9^, ^12^ and disagree with the studies of Rey et al.^43^ and Baraldi et al.,^44^ which did not observe such association.

Regarding the DMAH group, significant differences were observed at the frequencies of 500, 2 kHz and 3 kHz when compared to its control, as well as a trend to a statistically significant difference at 4 kHz and 6 kHz, indicating a significantly higher annual increase in auditory thresholds at these frequencies in the DMAH group. Studies that evaluated hearing in diabetic and hypertensive individuals obtained controversial results: Jorgensen and Buch^22^ did not observe any influence of these variables on hearing, whereas Duck et al.^45^ found such association. Oron et al.^46^ also observed that cardiovascular risk factors (including diabetes and hypertension) seem to have an effect on hearing, although there was no direct and causal correlation.

If we analyze the increase in auditory thresholds during the study period in the three groups, we can observe that the DM group, when compared to its control, did not show threshold worsening; the AH Group, when compared to its control, showed higher thresholds at the high frequencies and the DMAH Group, compared to its control, showed worse thresholds for low, medium and high frequencies. Additionally, when comparing the evolution of the three study groups (DM, AH and DMAH), it was observed that the mean annual increase is higher in the AH group, followed by the DMAH and, finally, the DM group.

One of the hypotheses for this finding could be related to the longer duration of the disease in the AH group. However, it is important to note that DM and SAH are diseases that may be asymptomatic and, therefore, the time of disease onset may be longer than that reported.^47^

Some studies have investigated the influence of disease duration on hearing threshold worsening. Regarding arterial hypertension, Esparza et al.^11^ studied a group with a mean disease diagnosis of 4 years and, even with that short time since the onset of disease, when compared to the present study they observed cochlear dysfunction in the individuals with SAH. Agarwal et al.^48^ evaluated distinct groups, divided into three different degrees of hypertension (by pressure level); the mean duration of the disease was 3.7 years for Grade 1 Group; 5.4 years for Grade 2 Group and 9.0 years for Grade 3 Group, with the latter group showing the worst auditory thresholds. It should be emphasized that the difference in time of hypertension duration observed in the different studies could influence the audiological results if we consider this pathology can cause microcirculatory insufficiency, which may result in greater or lesser deterioration of the peripheral auditory system.^49^, ^50^

In relation to DM, Sunkum and Pingile^19^ and Özel et al.^41^ found a positive association between diabetes duration and hearing loss, while Akinpelu et al.^35^ did not find a statistically significant association between diabetes duration and disease progression. However, the studied population, the methods employed and disease duration varied widely between the different studies, which may influence the observed results and explain these contrasting findings. Thus, this association between hearing loss and diabetes duration remains controversial.^41^

As previously mentioned, the groups studied in the present investigation differed with respect to the time since diagnosis (AH, DM and DMAH). However, our objective was not to correlate disease duration with auditory acuity worsening, but rather to verify, in the study follow-up period, which pathology would have the greatest influence on auditory thresholds. It should be emphasized that, to establish a correlation between time of diagnosis and hearing threshold worsening, another study design would be necessary, for instance, by comparing the auditory thresholds of groups with different disease duration and analyzing whether the auditory threshold worsening occurred non-linearly.

However, we cannot ignore the possible influence of disease duration, since, in the present study, we observed that in the study group with a longer disease duration, worsening of the auditory thresholds was more evident during the follow-up and that the hearing thresholds increased in hypertensive individuals at a higher rate than in the control group, even 16 years after diagnosis.

### Study limitations and potentials

It is important to note that the sample size of each study group was small and, perhaps, if comparisons were performed with larger groups, the differences between the auditory thresholds could be more evident. However, in the assessed age range it is difficult to find individuals that have only the assessed clinical conditions (DM and/or SAH).

Additionally, it is important to observe that the progression of auditory thresholds was measured linearly over a given period of time, without correlating hearing loss with disease duration, which would also require a larger sample.

It is noteworthy that the present study performed a complete auditory evaluation, as well as clinical and blood tests, which gives the findings a greater precision regarding the inclusion of individuals with AH and/or DM.

## Conclusion

It was observed that, when comparing the initial and final audiological assessments, the mean annual increase in auditory thresholds was higher in the AH group, followed by the DMAH and, finally, by the DM group, suggesting that among the studied conditions, arterial hypertension seems be the one that had the greatest influence on hearing. Regarding the most affected frequencies, it was observed that, for the AH group, the highest frequencies were most often affected, while for the DMAH group medium and high frequencies were the most affected ones.

## Funding

Fundação de Amparo à Pesquisa do Estado de São Paulo – 10.13039/501100001807FAPESP, process number 2013/05589-2.

## Conflicts of interest

The authors declare no conflicts of interest.
